# Post-esophagectomy Symptomatic Dunbar Syndrome: A rare diagnosis of abdominal pain after surgery

**DOI:** 10.1016/j.ijscr.2020.02.065

**Published:** 2020-03-07

**Authors:** Flavio Roberto Takeda, George Felipe Bezerra Darce, Lucas Faraco Sobrado, Luisa Leitão de Faria, Francisco Tustumi, Rubens Antonio Aissar Sallum, Manoel de Souza Rocha, Ulysses Ribeiro, Ivan Cecconello

**Affiliations:** a Department of Gastroenterology, Digestive Surgery Division, University of São Paulo Medical School, São Paulo, Brazil; b Department of Radiology, University of São Paulo Medical School, São Paulo, Brazil

**Keywords:** Esophagectomy, Abdominal pain, Median arcuate ligament, Dunbar syndrome, Angiotomography

## Abstract

**Introduction:**

Dunbar syndrome is a rare anatomical abnormality characterized by the extrinsic compression of the celiac trunk by the median arcuate ligament (MAL). Though it is rarely misdiagnosed, the clinical diagnosis may be difficult, especially after complex visceral surgery such as esophagectomy.

**Presentation of case:**

A 62-year-old male patient with a squamous cell carcinoma of the distal esophagus, placed under trimodal treatment (chemotherapy, radiotherapy followed by hybrid minimal invasive 2-field esophagectomy) presented with abdominal pain refractory to analgesics, anti-spasmodic, opioids, and neuronal celiac plexus ablation in the late post-operative period. He was diagnosed with extrinsic celiac trunk compression based on abdominal angiotomography findings. Retrospectively, similar images were found in conventional abdominal tomography at pre-operative staging, but this time, the patient had only dysphagia. After surgical treatment of MAL, the patient had total relief of pain and symptoms.

**Discussion:**

Abdominal pain after complex surgical procedures is very frequent and its investigation is mandatory, even more after refractory clinical management. Dunbar syndrome is related to ambiguous abdominal pain. It is uncommon and its diagnosis with angiotomography is accessible.

**Conclusion:**

Vascular disorders should be investigated in cases of abdominal pain after complex surgical procedures.

## Highlights

• Median Arcuate Ligament syndrome (MALS) is also known as Durban syndrome.
• MALS is a rare condition.
• MALS must be considered in case of refractory post-surgical abdominal pain.
• Angiotomography is accessible and reliable for making the diagnosis.

## 1 Introduction

Esophagectomy with lymphadenectomy is a technically challenging surgery, which currently has a low mortality rate and a high morbidity rate [1]. In late follow-up, dyspepsia is a frequent problem, and the most common hypotheses are gastroduodenal reflux, stenosis, and delayed emptying of the stomach [2]. The investigation of this condition is laborious and vascular pathologies should be recognized.

Low abdominal implantation of the median arcuate ligament (MAL) is a rare condition. When this is associated with celiac trunk (CT) compression and abdominal pain, it characterizes the Dunbar Syndrome [3].

Asymptomatic patients with low implantation of MAL may have changes in vascular dynamics after esophagectomy, due to the ligation of vascular trunks and may evolve with ischemic symptoms.

This case has been reported in line with the SCARE [4] criteria and it aims to describe the case of a patient with low abdominal implantation of MAL, which was previously asymptomatic, who developed celiac trunk compression-related symptoms post-esophagectomy with gastric tube reconstruction.

## 2 Presentation of case

A 62-year-old male patient with progressive dysphagia and weight loss was diagnosed with distal squamous cell carcinoma of the esophagus staged as T3N2 pre-operatively. The CT tomography revealed a discreet compression though he was asymptomatic.

He was placed on trimodal therapy (chemotherapy, radiotherapy followed by hybrid minimal invasive two fields esophagectomy with cervical gastroplasty – McKeown procedure). On follow-up, he developed postprandial dysphagia, vomiting, and epigastric pain. He was initially treated with prokinetics and proton pump inhibitors, but his symptoms were not relief.

**Fig. 1:**
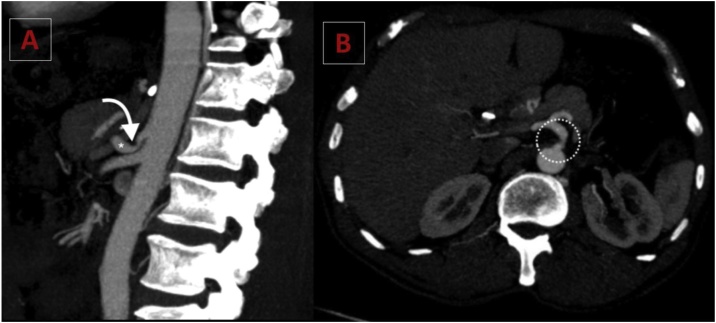
(1A) Sagittal maximum intensity projection (MIP) CT angiogram demonstrates the narrowing of the proximal celiac axis *(curved arrow)* caused by the median arcuate ligament compression and a poststenotic dilatation *(asterisk)*, creating a "hooked" appearance which is characteristic of the syndrome. Note the absence of atherosclerosis. (1B) Axial maximum intensity projection CT image shows the prominent collateral vessel and dilatation of the gastroduodenal artery (a common collateral pathway seen in patients with celiac axis stenosis).

**Fig. 2:**
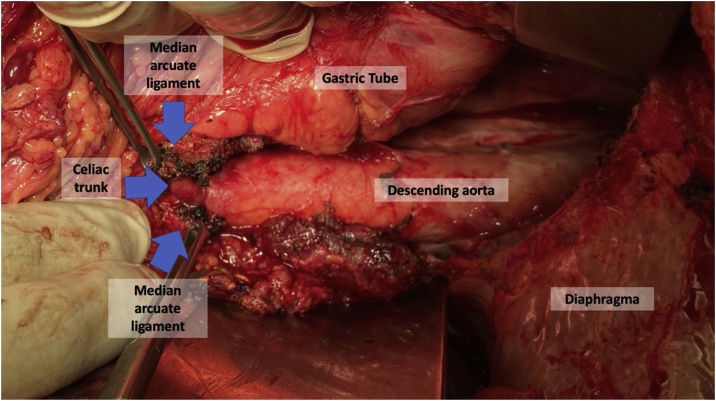
Intra-operative findings: Median arcuate ligament opened closed to celiac trunk. And also visualized the gastric tube, descending aorta e diaphragma.

A complete medical examination was performed, including upper endoscopy and esophagogram. However, abdominal computed tomography suggested compression of the CT by the arcuate ligament of the diaphragm, while other results were normal. Angiotomography (Fig. 1A and B) and Doppler ultrasound confirmed the findings which were present pre-operatively. An exploratory laparotomy and interruption (opening) of the arcuate ligament were performed (Fig. 2), which resulted in the elimination of extrinsic compression.

**Fig. 3:**
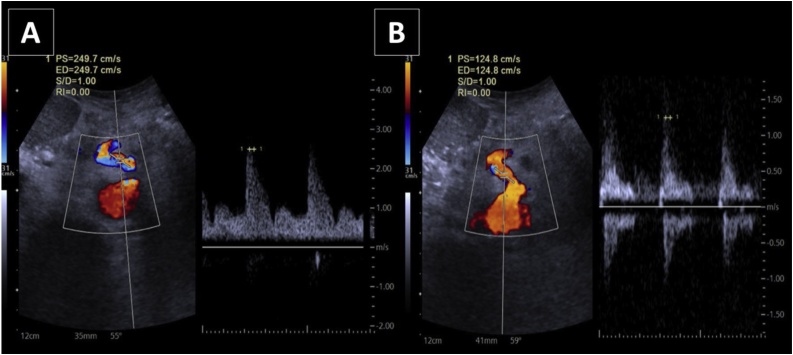
Spectral Doppler US with patient in supine decubitus, during inspiration (3A) and expiration (3B) at the narrower point of the celiac axis color aliasing point. Significantly elevated peak systolic velocity (249.7 cm/s) is seen on expiration with aliasing artifact at color-Doppler mode, 2 times greater than the velocity seen on inspiration (124.8 cm/s).

**Fig. 4:**
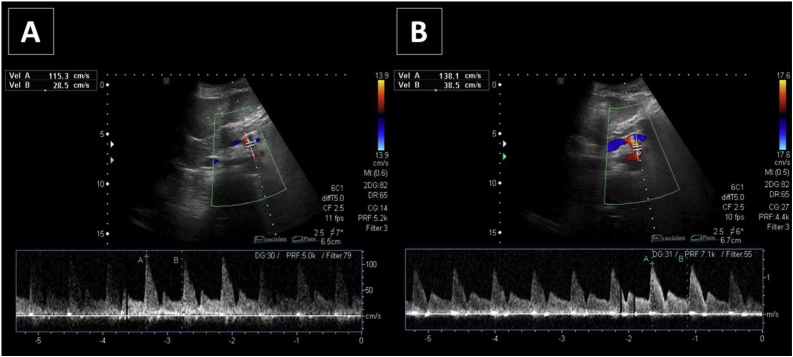
Postoperative spectral Doppler US with patient in supine decubitus, during inspiration (4A) and expiration (4B), showing the change in the peak systolic velocity during expiration after surgery, that became normal (138.1 cm/s).

In this case, Doppler ultrasound (US) detected the color aliasing in the proximal lumen of the celiac trunk during the expiratory phase with a significant increase of the peak systolic velocity (249.7 cm/s), 2 times greater than the velocity seen on inspiration (124.8 cm/s) (Fig. 3A and B). According to literature, a peak systolic velocity (PSV) over 200 cm/s during expiratory phase or a ratio more than 3:1 of PSV of celiac artery to aorta in expiratory phase is a Doppler criterion for diagnosis of Dunbar syndrome [[5], [6], [7]]. Moneta et al. [5] found that PSV values greater than 200 cm/sec defined >70% diameter-reducing stenosis of the celiac artery. The accuracy of this cut-off value in the diagnosis of celiac artery stenosis was 82%. Postoperative Doppler US performed 3 weeks after surgery (Fig. 4A and B) revealed normal velocity at the celiac artery origin during either inspiration (115.3 cm/s) or expiration (138.1 cm/s).

**Fig. 5:**
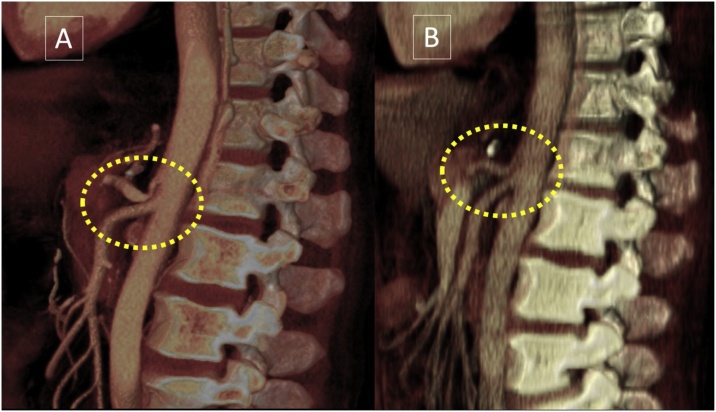
(A) Preoperative sagital 3D volume-rendered image demonstrates characteristic hooked appearance of the stenotic celiac axis. (B) Postoperative sagittal 3D volume-rendered image shows the resolution of the narrowing of the proximal celiac axis and the poststenotic dilatatation.

The postoperative course was uneventful and follow-up imaging, including angiotomography and Doppler ultrasound, showed decompression of the CT (Fig. 5A and B; supplementary material). After 3 months of follow-up the patient remained free of any symptoms.

## 3 Discussion

MAL is a fibrous arch formed at the base of the diaphragm at the level of the twelfth thoracic vertebra, where the left and right diaphragmatic pillars join. It forms the anterior face of the aortic hiatus, through which the aorta, thoracic duct, and azygos vein pass. MAL is usually in contact with the aorta above the origin of the celiac trunk [3].

In 1965, Dunbar related this anatomical anomaly to digestive symptoms and described it as a part of a clinical syndrome for the first time, which later became known as arcuate ligament syndrome or Dunbar syndrome. It has a prevalence of 2 per 100,000 cases of abdominal pain. It affects mostly women (Ratio 3:1), the thin, and people between 18 and 30 years.

The pathophysiology of the disease consists of extrinsic CT compression by the abnormally low-implanted MAL. Compression worsens with expiration as the diaphragm moves caudally, resulting in worsening of CT stenosis. This compression results in reduced CT blood flow, visceral ischemia, and postprandial abdominal pain [5].

Overstimulation of the celiac ganglion is also believed to result in chronic pain in these patients. Some patients may experience changes in bowel habits secondary to midgut ischemia due to the diversion of collateral blood flow from the superior mesenteric artery to the CT [5].

Similar to the present case, asymptomatic patients with evidence of celiac trunk compression have achieved homeostasis via collateral arterial branches [6].

We performed esophagectomy using a gastric tube reconstruction with ligation of the left gastric artery and the left gastroepiploic artery. After this procedure, the branches responsible for the vascular supply of the constructed gastric reservoir were; the right gastroepiploic artery a branch of the gastroduodenal artery, and the right gastric artery, a branch of the hepatic artery itself, which is a direct branch of CT [9]. Thus, a vascular compromise of CT could justify the episodes of mesenteric angina affecting the neo-stomach, manifesting as postprandial abdominal pain.

When symptomatic, MALS usually causes postprandial epigastric pain (80%), nausea (9.7%), weight loss (48%), diarrhea (7.5%), and an epigastric murmur may be present on auscultation [5].

Owing to nonspecific symptoms, the diagnosis usually made by excluding other conditions. The medical examination of these patients includes abdominal ultrasonography with Doppler study of mesenteric vessels and the CT, abdominal computed tomography with vascular study (angiotomography) or angiography [5].

Patients with a history of esophagectomy with postoperative dyspepsia should initially be suspected for post-surgical complications such as gastroduodenal reflux or pyloric stenosis or delayed gastric tube emptying [7]. The recommendations for an initial investigation are upper digestive endoscopy and contrast examination of the esophagus, stomach, and duodenum. When normal, we proceed with computed tomography of the abdomen, which besides morphological alterations also allows the observation of vascular anatomical alterations, which can be further studied for functional evaluation with doppler ultrasonography [8].

In the past, angiography was considered the gold standard for the diagnosis of the condition. In the sagittal section, it reveals focal stenosis of the CT associated with post-stenotic dilation and increased collateral vascularization from the superior mesenteric artery [6].

Currently, thin-cut multiple-channel angiotomography associated with three-dimensional reconstruction has become the best method for obtaining high-resolution images of the aorta and its branches. CT angiography, especially during expiration, has a high accuracy in identifying the syndrome. Moreover, this method also allows visualization not only of the stenosed vessel but also of the underlying median arcuate ligament and adjacent tissues. Angiotomography is also important to exclude the presence of calcifications in the CT which is an important cause of arterial stenosis [5].

Treatment options include video laparoscopy or laparotomy with sectioning of the MAL and celiac plexus fibers, as well as with the percutaneous transluminal angioplasty approach. Persistent vessel deformity or pressure gradient after the decompression procedure would be indications of approach with vascular reconstruction. The persistence of symptoms due to incomplete CT release or restenosis has been successfully treated by angioplasty with endovascular stenting within three months.

## 4 Conclusion

Vascular disorders should be investigated in cases of refractory abdominal pain after complex surgical procedures.

## Declaration of Competing Interest

The authors declare no conflict of interest.

## Sources of funding

The authors received no specific funding for this work.

## Ethical approval

Ethical approval exemption was given for this study.

## Consent

Written informed consent was obtained from the patient for publication of this case report and accompanying images. A copy of the written consent is available for review by the Editor-in-Chief of this journal on request.

## Author’s contribution

Flavio Roberto Takeda: Conceptualization, writing, formal analysis.

George Darce: writing – review and editing.

Lucas Sobrado: writing – review and editing.

Luisa Faria: writing – review and editing radiological images.

Francisco Tustumi: writing – review and editing.

Rubens Sallum: writing and Supervision.

Manoel de Souza Rocha: writing – review and editing radiological images.

Ulysses Ribeiro: writing and Supervision.

Ivan Cecconello: Supervision.

## Registration of research studies

Not applicable.

## Guarantor

Flávio Roberto Takeda.

## Provenance and peer review

Not commissioned, externally peer-reviewed.
